# Application of CRISPR-Cas9 Gene Editing for HIV Host Factor Discovery and Validation

**DOI:** 10.3390/pathogens11080891

**Published:** 2022-08-09

**Authors:** William J. Cisneros, Daphne Cornish, Judd F. Hultquist

**Affiliations:** 1 Division of Infectious Diseases, Northwestern University Feinberg School of Medicine, Chicago, IL 60611, USA; 2 Center for Pathogen Genomics and Microbial Evolution, Northwestern University Havey Institute for Global Health, Chicago, IL 60611, USA

**Keywords:** HIV, host factors, CRISPR-Cas9, screening, gene editing

## Abstract

Human Immunodeficiency Virus (HIV) interacts with a wide array of host factors at each stage of its lifecycle to facilitate replication and circumvent the immune response. Identification and characterization of these host factors is critical for elucidating the mechanism of viral replication and for developing next-generation HIV-1 therapeutic and curative strategies. Recent advances in CRISPR-Cas9-based genome engineering approaches have provided researchers with an assortment of new, valuable tools for host factor discovery and interrogation. Genome-wide screening in a variety of in vitro cell models has helped define the critical host factors that play a role in various cellular and biological contexts. Targeted manipulation of specific host factors by CRISPR-Cas9-mediated gene knock-out, overexpression, and/or directed repair have furthermore allowed for target validation in primary cell models and mechanistic inquiry through hypothesis-based testing. In this review, we summarize several CRISPR-based screening strategies for the identification of HIV-1 host factors and highlight how CRISPR-Cas9 approaches have been used to elucidate the molecular mechanisms of viral replication and host response. Finally, we examine promising new technologies in the CRISPR field and how these may be applied to address critical questions in HIV-1 biology going forward.

## 1. Introduction

All viruses, from the simplest to the most complex, rely on host cell machinery for replication and immune evasion. Human Immunodeficiency Virus (HIV) is a lentivirus that replicates by reverse transcribing its roughly 10 kb RNA genome into DNA, which is then integrated into the human genome. This integrated provirus is transcribed, and the resultant RNA transcripts are translated and processed to produce the 15 viral proteins required for successful production of new, infectious virions. Given this limited repertoire of viral proteins, every step of the HIV lifecycle—including viral entry, reverse transcription, cytoplasmic transport, uncoating, nuclear entry, proviral integration, transcription, translation, assembly and egress—is mediated by, and dependent on, host proteins, nucleic acids, and metabolites collectively known as host factors [1]. Host factors are broadly categorized as either dependency factors that promote viral replication or as restriction factors that inhibit viral replication and must be somehow counteracted for successful replication of the virus [2,3].

Host factors play critical roles in viral replication and pathogenesis, and so have emerged as promising targets for the development of next-generation therapeutics [1,4]. For example, the FDA-approved antiretroviral drug Maraviroc functions to inhibit viral entry by binding to the HIV co-receptor CCR5, preventing binding of HIV envelope and inhibiting viral entry [5,6]. A variety of host factors have also been described to facilitate or impede the establishment and maintenance of HIV latency. The persistence of transcriptionally restricted proviruses in long-lived, latent cellular reservoirs is one of the primary barriers to the development of a functional HIV cure. Consequently, a number of host factor-directed therapies have been explored in recent years as a means to manipulate the latent reservoir [7,8]. 

**Figure 1:**
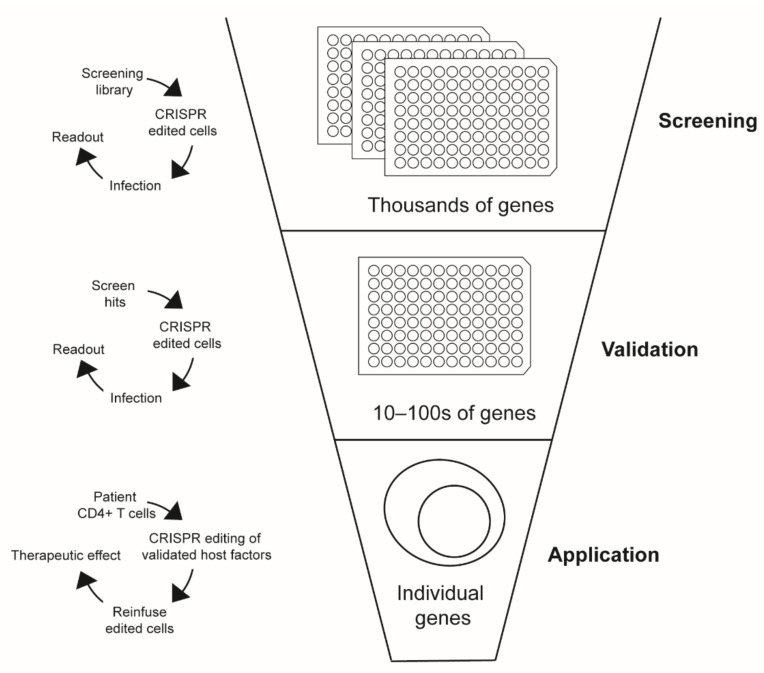
**Use of CRISPR-Cas9 in Host Factor Screening, Validation, and Therapeutic Applications.** CRISPR-Cas9 serves as a valuable tool in host factor discovery, validation, and therapeutic intervention. As a tool for in vitro studies, CRISPR-Cas9 enables researchers to conduct high-throughput genetic screens of thousands of potential host factors in cells lines and primary cells. After potential host factors are identified through genetic screens, CRISPR-Cas9 can be used in follow-up studies to validate and determine the mechanism of these host factors. CRISPR-Cas9 is also being investigated as a therapeutic strategy against infection.

Given their vital functions in HIV biology and their potential use as next-generation therapeutic or curative targets, the identification and functional characterization of HIV host factors remains a high priority goal in HIV research. Both of these goals have been significantly advanced by the maturation of CRISPR-Cas9 gene editing technologies for the efficient, targeted manipulation of human gene expression [9,10]. The development of high-throughput arrayed and pooled screening platforms has enabled the identification of new host factors in a variety of different model systems and cellular contexts. The broad applicability of CRISPR-Cas9 gene editing to a variety of human cell lines, animal models, and even primary human cell types has likewise facilitated discoveries in host factor mechanism and function. Furthermore, the use of this technology to render patient cells resistant to HIV infection ex vivo has stimulated interest in understanding how such advances could be translated to in vivo applications. In this review, we will outline the use of CRISPR-Cas9 gene editing in host factor discovery and validation towards the development of next-generation host-directed therapeutic strategies (Figure 1).

## 2. Genetic Screens and the Identification of HIV-1 Host Factors

HIV replication is impacted by a broad array of host factors that vary by cell type, cellular environment, and viral genotype [1]. These factors may act through direct interactions with viral proteins and/or nucleic acids or through indirect interactions as regulatory factors or as members of larger complexes. Many different approaches have been used to identify these host factors using either physical or functional screening approaches. Screens for physically interacting host factors have been conducted through a variety of approaches including yeast-two-hybrid library screening and mass spectrometry following whole virion precipitation or viral protein/nucleic acid purification [11]. Screens for functionally relevant host factors have likewise been conducted through many approaches including transcriptional profiling and genetic screening following gene perturbation or overexpression [12]. All of these approaches have their own distinct advantages and limitations; for the purposes of this discussion, we will be focusing on genetic screens.

**Figure 2:**
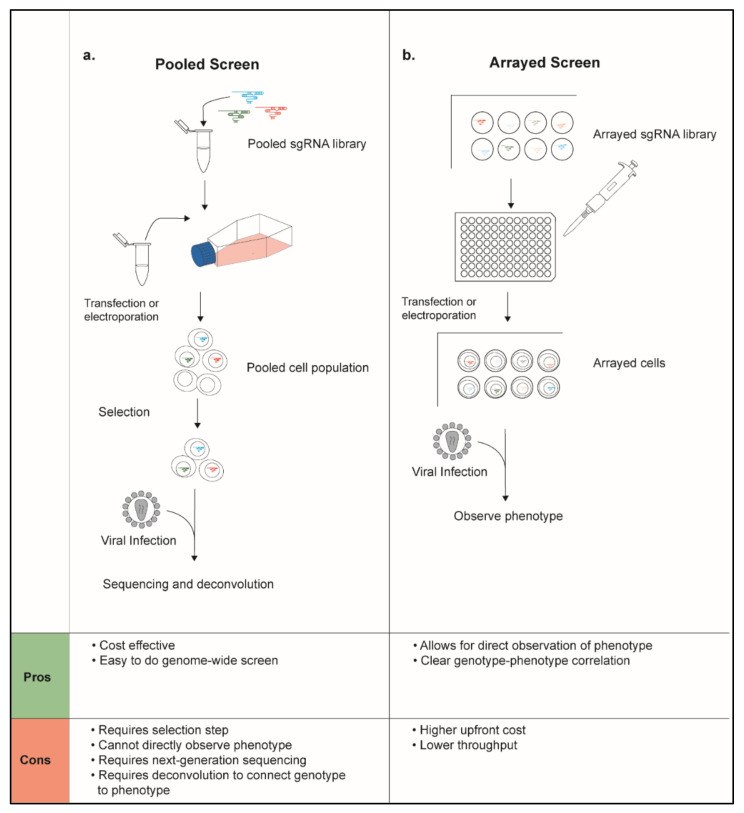
**Pooled versus Arrayed CRISPR-Cas9 Screens in Host Factor Discovery.** Genetic screens aimed at host factor discovery can be conducted in a pooled or an arrayed format. (**a**) Pooled libraries of sgRNAs are delivered to cells, resulting in a population of cells with different genetic perturbations. Cells are then selected based on successful genetic editing. Following viral infection, cells undergo next-generation sequencing to identify screen hits. (**b**) In arrayed screens, CRISPR-Cas9 reagents are synthesized to target a single gene in each well of multi-well plates. Following infection, the phenotype of interest for the screen can be directly observed in each well.

The principle behind a genetic screen for viral host factors is as follows: the genetic perturbation of a functionally relevant host gene will result in a significant change in a quantifiable viral phenotype. These perturbations can be driven by systematic mutagenesis, RNA interference, or through targeted gene editing approaches such as CRISPR-Cas9 [13,14]. The phenotypic readout can be tailored to examine the effect of genetic manipulation on a specific viral protein or protein function, a related host cell process, or may even be multiplexed such that several phenotypes are monitored in a single experiment [12,15]. The most common readout leveraged in HIV host factor screens has been proviral gene expression following integration as measured by expression of a reporter gene (i.e., GFP, luciferase, etc.) that is either in the virus or in the host cell genome and induced by infection. Depending on the complexity of the readout, these screens can be expanded to cover the majority of the genes in the genome or may be limited to a small subset of genes that are related as shown by past experimental evidence, form, or function. Complexity of the readout and the size of the library will dictate whether the screen should be conducted in a pooled or arrayed format (Figure 2).

In a pooled screen, all genetic perturbations are carried out in a single population of cells [16]. This cell pool is ultimately sorted or separated based on a given phenotypic readout. The perturbations responsible for the resulting phenotype are often determined by the deep sequencing of a perturbation-specific barcode. These are often delivered using lentiviral vectors that stably integrate their payload into the target cell genome to allow for continuous expression of the perturbing agent (i.e., a short hairpin RNA (shRNA) or CRISPR single guide RNA (sgRNA)), co-delivery of a selection marker, and stable insertion of an identifying barcode. These libraries generally include multiple independent non-targeting controls and multiple independent shRNA or sgRNA targeting each gene of interest. Most libraries include 4–5 shRNA or sgRNA per gene, though some ultracomplex libraries may have up to 25 independent perturbants per gene. These libraries are delivered to cells via lentiviral transduction at a low multiplicity of infection (MOI) to ensure that only a single genetic perturbation occurs per cell. Following library delivery and selection for transduced cells, the resultant pool of cells is subject to selective pressure and phenotypically sorted. In the case of HIV host factor screening, a common phenotypic readout may be cell challenge with a GFP-containing reporter virus followed by sorting for infected (GFP+) versus uninfected (GFP−) cells. Barcodes can then be amplified from each population of cells and quantified by next-generation sequencing to identify candidate host factors.

Pooled screens can be exceptionally powerful tools for initial discovery of genes of interest [17]. Pre-existing libraries and protocols lower the barrier to entry, while the pooled format simplifies the workflow. If each perturbant is represented 1000-fold in a population of cells, genome-wide screens can be done in a population of 100–150 million cells, making the approach relatively cost-effective. Nevertheless, there exist a number of drawbacks to a pooled screening approach that can limit applicability. First, the selective readout applied is rarely perfectly discriminatory and multiple rounds of selection may be required to identify relevant factors. The strength of each selective pressure must be carefully weighed to ensure phenotypic discrimination, but to prevent population bottlenecking. Multiple biological replicates are required for robust scoring. Second, biological confounders may uncover several circumstantial hits that are not directly involved in the process of interest. For this reason, pooled screens often require extensive follow-up and validation after initial hit identification. Third, given that no information is collected on perturbation efficacy, biological insight is usually limited to positive hits. In other words, a lack of a phenotype cannot be assumed to be due to lack of biological function but may be due to a technical limitation in perturbation or selection.

As opposed to pooled screens, arrayed screens are formatted such that every gene target or perturbation occurs in an independent well [16]. Since there is only one target per well, the correlation between genotype and phenotype in an arrayed screen is immediately clear and does not require deconvolution. This enables a broader array of perturbation delivery methods that do not involve the stable integration of a barcode, such as transfection of small interfering RNA (siRNA) or electroporation of CRISPR-Cas9 ribonucleoprotein complexes (crRNPs). This format generally is more accessible for a broader selection of cell types and a broader selection of downstream readouts, including fluorescence and cell imaging-based platforms. In addition, arrayed screens are easy to adapt to multiplex readouts, including direct measurement of perturbation efficiency, cell viability, and even cell-extrinsic effects. Nevertheless, despite these advantages, there are several limitations to this approach. The number of genes that can be efficiently screened in an arrayed format is lower than in a pooled screen, requiring a subset of genes be predetermined for analysis. Larger arrayed screens can be cost prohibitive and may require access to robotics and automated instrumentation. Given that these screens are generally more targeted, they often require the custom synthesis of an arrayed library, which can be costly and time-consuming. Finally, the analysis of most arrayed readouts in high-throughput screening typically requires additional computational or biostatistical expertise as it is often not amenable to analysis by automated platforms.

Despite these many challenges, several targeted and genome-wide screens, both arrayed and pooled, have been conducted for the identification of HIV host factors (Table 1). These have relied on a variety of perturbation approaches, cell types, and viral strains, but all have resulted in the identification of new host factors.

### 2.1. RNAi HIV-1 Host Factor Screens

Early efforts to identify HIV-1 host factors via genetic screening employed high-throughput RNA interference (RNAi) technologies to conduct loss-of-function screens. RNAi suppresses the expression of genes via introduction of short (20–25 nucleotides) RNA species that are complementary to an mRNA target. Binding results in inhibition of mRNA translation and/or degradation of the mRNA target, silencing gene expression [32]. These are generally delivered in the form of double-stranded short interfering RNA (siRNA) or as short hairpin RNA (shRNA) that are then processed by host cell machinery and incorporated into RNA-induced silencing complexes for effector function. RNAi has been widely used for the disruption of gene expression in cell culture and has been developed into several genome-wide screening platforms.

The first three genome-wide screens for HIV host factors by Brass et al., König et al., and Zhou et al. used an arrayed siRNA screening approach to systematically knock-down genes in TZM-bl, HEK293T, and HeLa cells, respectively [19,20,21]. These cells were then challenged with lab-adapted HIV-1 strains with various measures of infection used as a readout (Table 1). Each screen identified hundreds of candidate host factors suggested to play a role in HIV-1 infection, including several critical host factors involved in cytoplasmic transport and nuclear entry such as TNPO3, NUP153, and NUP358 [19,20,21]. A later screen by Yeung et al. took a different approach, leveraging stably expressed shRNA to conduct a pooled screen in Jurkat T cells [18]. This approach similarly identified hundreds of candidate host factors that act at various steps of the HIV-1 replication cycle [18].

While these landmark studies revealed a wealth of information regarding host proteins critical to HIV-1 pathogenesis, a meta-analysis of these screens revealed that there was little concordance between the candidates discovered for each screen [33]. Comparison of the host factors identified by these RNAi screens revealed less than 7% overlap of hits among any two of these four screens, with no hits shared by all of them [33]. Furthermore, there was often little concordance between the screen data and previously described host factors in the literature. This lack of consensus has been attributed to a wide variety of factors including off-target effects of the RNAi technologies, the use of different (and often contrived) cell line models for infection, the use of different viral strains and infection readouts, and the use of different scoring strategies and statistical methodologies [15]. While examination of the gene candidates between these screens at the level of molecular pathways and networks revealed more concordance than initially assumed, and while each study did reveal and mechanistically validate new host factors, the limitations of using these approaches for comprehensive host factor identification became more apparent.

Following up on the poor concordance observed in these initial results from the HIV-1 host factor RNAi screens, Zhu et al. suggested a number of methods to increase the robustness of such screens, including the use of multiple orthologous RNAi oligonucleotides and standardized downstream bioinformatics analysis to mitigate issues with high false positives, false negatives, and off-target effects [34]. Since then, a number of groups have employed next-generation, genome-wide, RNAi-based pooled screens to identify HIV-1 dependency factors, HIV-1 restriction factors, and host factors associated with the reversal of transcriptional silencing in latency models [35,36,37,38]. While these have likewise led to the identification of important host factors, inherent limitations in RNAi technology have imposed some unavoidable drawbacks in this screening approach. As RNAi targets mRNA and does not wholly ablate gene expression, the targeted gene is usually expressed at a residual level after knock-down [32]. Incomplete knock-down of genes can result in lowered sensitivity in loss-of-function screens, making it difficult to identify host factors required at only low cellular levels (i.e., LEDGF) [15]. Off-target effects can likewise complicate screen design and interpretation, requiring the use of multiple, overlapping siRNAs for a given gene to prevent false positives and necessitating extensive validation of screen hits using orthogonal reagents on the back end [39].

### 2.2. CRISPR-Cas9 Gene Editing Technologies

The advent of CRISPR-Cas9 genome editing has led to substantial advances in genetic screening methods. CRISPR-Cas9 gene editing technology is adapted from a naturally occurring process in the adaptive immune system of bacteria and archaea [16,40,41,42]. Cas9 is a programmable nuclease that is targeted to a complementary site in the genome by an associated guide RNA. Cas9 induces a DNA double-stranded break at the target site, which can be subsequently repaired by the host DNA repair pathways. Imperfect repair through non-homologous end-joining often results in the generation of small insertions and deletions that lead to frameshift mutations in the gene and permanent knock-out of gene expression [43,44]. Several different delivery methods have since been developed for the editing of mammalian cells. Plasmid-based and lentiviral vector-based systems often allow for the cognate delivery of both the Cas9 protein and single guide RNA (sgRNA) in a selectable manner [40]. Alternately, delivery of in vitro synthesized CRISPR-Cas9 ribonucleoprotein complexes (crRNPs) by electroporation or transfection can allow for efficient and scarless editing of the genome without the need for cloning of the associated components [45]. All these methods result in direct, stable editing of the genome and complete ablation of gene expression, facilitating loss-of-function genetic screens.

CRISPR-Cas9 technology has since been adapted to elicit other genetic perturbations besides gene knock-out. For instance, CRISPR-Cas9 has been adapted to repress gene expression via CRISPR interference (CRISPRi) [46,47]. CRISPRi involves delivery of a catalytically dead Cas9 (dCas9) that lacks endonuclease activity, but that can still be targeted to and bind specific sites in the DNA. Binding of dCas9 can interfere with transcriptional elongation, RNA polymerase binding, as well as transcription factor binding to ultimately disrupt gene expression. The addition of potent transcriptional inhibitor domains to dCas9, such as the Kruppel associated box or KRAB domain, can further enhance transcriptional repression. Since this method of gene silencing does not involve cutting of the target DNA sequence, it can be engineered to be inducible, reversible, and titratable, providing some unique advantages compared to CRISPR knock-out (CRISPRko) approaches [48,49]. On the other hand, CRISPRi requires continual expression of dCas9 for transcriptional repression, limiting delivery methods, and may result in only partial repression, resulting in less sensitive phenotypic screening [48].

The CRISPR-Cas9 system can also be used to induce gene upregulation through CRISPR activation (CRISPRa). CRISPRa leverages a catalytically dead Cas9 fused to a transcriptional activation domain (such as the VP64 transactivator domain) to induce overexpression of target genes [50]. CRISPRa provides a unique tool for use in gain-of-function screening methods that have otherwise only been possible through exogenous expression via plasmid-based library.

CRISPR-Cas9 approaches have a few advantages over RNAi-mediated methods for high-throughput screening. In general, there are fewer off-target effects compared to RNAi, though the use of multiple independent guide RNA per gene target is still required for experimental rigor [16,41]. CRISPRko also allows for direct genome editing and thus leads to total ablation of gene expression, enhancing screen sensitivity and ensuring detection of factors required at only low abundance. Finally, the ability to leverage CRISPRko, CRISPRi, and CRISPRa-based screening technologies enables cross-comparison for robust candidate assessment. Given these advantages, a number of CRISPR-Cas9 screens for identifying HIV host factors have been executed in a variety of formats since the discovery and development of CRISPR-Cas9 (Table 1) [12].

### 2.3. CRISPR-Cas9 Pooled Screens for Identifying HIV Host Factors

One of the first genome-wide CRISPR screens for HIV host factors leveraged a pooled, lentiviral library of sgRNA containing nearly 10 sgRNA per gene targeting over 18,000 gene targets [22]. This screen by Park et al. utilized GXRCas9 cells, a CD4+ T cell line derived from CCRF-CEM human T lymphoblasts, which expressed GFP upon productive infection with HIV-1. Cells were serially challenged with HIV-1 and infected (GFP+) cells were removed from the cell pool by a combination of HIV-associated cell death and by flow sorting. The barcoded sgRNA were sequenced from the surviving cell pool to identify dependency factors. Surprisingly, only five genes were found to be significantly enriched: the receptor *CD4*, the coreceptor *CCR5* and three novel factors *TPST2*, *SLC35B2*, and *ALCAM* [22]. Both TPST2 and SLC35B2 were found to be important for CCR5 sulfination, and so contributed to cell entry, while ALCAM was found to contribute to cell-to-cell spread. One of the major limitations of this screening approach was that not all cells could be challenged with virus at once. Due to limitations in the multiplicity of infection (MOI) that could be achieved, multiple rounds of selection needed to be applied to ensure all cells received the selective pressure. This ultimately enriched for the most impactful perturbations, namely those that impacted cell entry and spread.

To overcome the inherent issue with MOI in screens that rely on HIV infection, Ohainle et al. developed a new CRISPR-Cas9 approach for pooled host factor screening that uses the virus as a readout instead of the cells [28]. Briefly, this group generated an sgRNA cassette (termed HIV-CRISPR) that will co-package *in trans* with budding HIV virions from infected cells. Thus, HIV viruses produced after infection of a given knock-out cell will carry the sgRNA from that cell as a marker. As a result, if a dependency factor is perturbed, fewer viruses with that sgRNA will be produced and if a restriction factor is perturbed, more viruses with that sgRNA will be produced [28,51]. In a proof-of-concept screen, the authors assembled a library targeting 1905 ISGs (termed the Packageable Interferon Stimulated Gene Knockout Assembly HIV or PIKA_HIV_ library) and assessed interferon-mediated inhibition of HIV-1 in the monocytic THP-1 cell line [28]. This screen identified fifteen ISGs which are involved in inhibiting HIV-1 as well as additional host dependency factors. A subsequent screen using the same method identified capsid-targeting HIV-1 restriction factors, including TRIM34 as a novel post-entry restriction factor that works in tandem with TRIM5α [29]. The HIV-CRISPR screening method eliminates considerations about MOI that are inherent to typical pooled host factor screens, is compatible with a wide variety of cell lines and HIV-1 strains, and can be used to assess both early and late steps in the viral lifecycle [51].

Notably, no genome-wide, arrayed screens for HIV host factors using CRISPR-Cas9 have yet been reported.

### 2.4. CRISPR Screens Exploring HIV-1 Latency

A number of CRISPR-Cas9 screens have also been employed to identify host factors involved in the establishment, maintenance, and reversal of HIV latency. This focus has been guided in part by curative strategies aimed at either reversing latency to facilitate killing of infected cells (“shock and kill”) or for inducing cells to enter a deep state of latency in which the virus remains completely dormant (“lock and block”) [52,53]. Rather than assessing impact of host factors on viral infection, latency screens have largely focused on the identification of host factors that either activate or inhibit proviral transcription in cell line models of HIV-1 latency [23,24,25].

To date, two pooled CRISPRko screens have been used to identify host factors that promote viral latency. These screens by Huang et al. and Krasnopolsky et al. utilized cell lines (J-Lat and Jurkat 2D10 cells, respectively) that harbor transcriptionally inhibited proviral transcripts containing a reporter gene. For these screens, the readout of interest is reactivation of the proviral sequence as determined by reporter activity. These screens uncovered a number of lead candidate host factors which promote viral latency, including MINA53 and ZNF304 [23,24]. In addition to CRISPRko screens, CRISPRi screening has also been used to identify host factors that may play a role in HIV latency. For example, Li et al. deployed a method termed Reiterative Enrichment and Authentication of CRISPRi Targets (or REACT), which leveraged an iterative approach of refining sgRNA libraries over multiple rounds of selection to identify host factors that contribute to latency [25]. The authors delivered a genome-wide lentiviral sgRNA library to the Jurkat 2D10 cell line model and selected for cells which reactivated proviral transcripts after knock-out. sgRNA sequences were amplified from the selected cells and subsequently used to create a new, enriched sgRNA library for a second round of screening. This process was repeated for five rounds with the goal of minimizing background signal arising from stochastic proviral reactivation events. This screen identified six candidate genes that promote latency reversal, three of which were subunits of the proteasome. A similar approach was applied in a subsequent study to identify host factors that promote HIV-1 latency, identifying a number of novel candidates including TMEM178A, FTSJ3, NICN, and the Integrator complex [26]. Most recently, Pederson et al. utilized CRISPRi to identify host factors that suppress HIV-1 reactivation in the context of latency [27]. This genome-wide screen employed four HIV-1-d6-GFP-infected Jurkat T cell clones, with each clone containing a reporter provirus integrated into actively transcribed genes [54,55]. Ultimately, the screen identified 18 host factors that contribute to HIV-1 silencing, including SLTM, SRRM2, and NCBP2. Though successful in identifying candidates, it is unclear how penetrant these host factors may be in promoting or enhancing HIV latency outside of the cell line models explored here.

### 2.5. Primary Cell CRISPR-Cas9 Screens

All of the genetic screens for HIV-1 host factors described above relied on the use of immortalized cell line models as these cells are easy to propagate in bulk and easy to manipulate. However, cell lines do not always recapitulate disease and infection-associated phenotypes in a way that translates to studies in humans [33]. Immortalization of cells can lead to changes in essential cell processes including stress responses and immune responses, all of which could alter host factor dependencies in a cell type specific manner [56]. As such, genetic screens usually require additional validation of hits in primary cell models. Park et al. provide one example where one of the five host factor candidates discovered in the cell line-based screen (*ALCAM*) did not validate in primary CD4+ T cells as it was not expressed in that cell type. To overcome this limitation, recent advances in genome editing technology and primary cell culture techniques have focused on expanding the use of CRISPR-Cas9 gene editing tools into diverse primary cell types.

The use of CRISPR-Cas9 to conduct HIV-1 host factor screens in primary cells was first reported by Hultquist et al. With this screening strategy, in vitro synthesized crRNPs are delivered to activated primary CD4+ T cells isolated from the blood of multiple independent donors by electroporation [57]. Though the crRNPs have a transient lifespan, a single pulse with an efficient guide is sufficient to yield near complete knock-out in a polyclonal pool. This allows for the efficient editing of primary CD4+ T cells in high-throughput arrayed format for host factor screening. After demonstrating proof-of-concept with a number of known host factors, the authors screened 45 genes associated with HIV-1 integrase in arrayed format and identified a number of candidate host factors which were reproducible across cells from multiple independent donors, including *GEMIN2*, *KPNA1*, *KPNA5*, and *XRCC6* [30]. This was later followed up in Hiatt et al., who used this approach to target over 400 putative host factors previously suggested to physically interact with HIV proteins [31]. Using amplicon sequencing to assess guide RNA efficacy, the authors were able to successfully knock-out over 350 of the targeted genes, identifying 86 putative host factors with functional roles in primary CD4+ T cells. This platform has since been expanded for use in primary human monocytes, though this approach has not yet been used for host factor screening. Likewise, a pooled version of genome-wide CRISPR screening in primary human T cells has been reported but has not yet been used for HIV host factor screening [58].

### 2.6. Limitations of CRISPR-Cas9 Screens

While CRISPR-Cas9 gene editing has revolutionized our ability to manipulate the human genome, there are still a number of limitations inherent to the technology that must be considered during screen design and interpretation. For instance, some genetic loci are not readily targetable by CRISPR-Cas9, potentially due to inaccessible chromatin structure or genome architecture at the target site [59]. Thus, it is important to validate editing efficiency of guides prior to assigning confidence in any given hit. Furthermore, ablation of essential genes via CRISPR-Cas9 can lead to cell death, resulting in phenotype masking due to outgrowth of unedited cells or cells with in-frame repairs. This problem can be mitigated by monitoring of cell viability and population count. This can be especially problematic when using non-selectable editing methods such as crRNP electroporation. These approaches result in a polyclonal population of cells in which some cells remain unedited, others have heterozygous knockout of the targeted gene, and others have homozygous knockout. The efficiency of a guide RNA will determine the proportion of cells that make each of these groups within the polyclonal cell population, which will in turn determine the degree to which a phenotype is displayed in a genetic screen [57].

Additional challenges for CRISPR-Cas9 HIV host factor screens arise in considerations of scale and scope. Most pooled CRISPR-Cas9 screens utilizing lentiviral delivery of CRISPR libraries require initial transduction at a low multiplicity of infection (MOI) in order to ensure delivery of a single guide RNA to each cell, reducing the false-positive discovery rate [42]. However, this generally requires batch culture of a very large number of cells, which can complicate efforts with certain cell types, including primary cells. After library generation, the initial library must be sequenced to ensure all comparisons post-selection are made relative to the parental population or relative to an unselected control. The selective pressure applied must then be carefully considered to both be amenable to the appropriate scale as well as to avoid overly stringent selection that may induce population bottlenecking and false positives. In screens involving HIV challenge, viral titer must be carefully selected and may require multiple rounds of challenge to ensure sufficient discrimination between susceptible and resistant populations.

Given that arrayed screens require a separate population of cells for each genetic perturbation, these screens can likewise require a vast amount of resources when applied at a genome-wide level [14]. Even more resources are required in the case of arrayed screens using primary cells, as multiple replicates may be needed to conduct the screens using cells from independent donors to account for donor-to-donor variability. In cases where genome-wide screens are impractical, these screening approaches may be employed using a narrower, targeted set of genes based on previous literature, data, or a phenotype of interest [31].

### 2.7. Future Directions for CRISPR-Cas9 Host Factor Screens

CRISPR-Cas9 genome editing has yet to be utilized to its full potential in the context of HIV-1 host factor screens. To date, only a single pooled CRISPR-Cas9 screen for host factors that act during active HIV-1 replication has been reported [22]. Furthermore, no genome-wide, arrayed CRISPR screen for HIV-1 host factors has been utilized to date. Until additional CRISPR-Cas9 based screens are performed and published, the reproducibility of such screening results remains unclear. Individual hit validation and comparison to benchmarks from the literature will be required to assess screen rigor. Of the genome-wide screens described, all have relied on cell line models of infection (Table 1). Other approaches to CRISPR-Cas9 genome editing, which allow for gene overexpression (CRISPRa) and transient repression of gene expression (CRISPRi), remain largely unexplored in the context of HIV-1 host factor screens. CRISPRa has been used for genetic screens to identify druggable targets for a variety of diseases but has yet to be adapted for use in HIV-1 host factor screens [49,50]. CRISPRa could be used for gain-of-function HIV-1 host factor screens as a complement to the loss-of-function screens which have already been performed. CRISPRi has only been used in the context of HIV-1 host factors screens in latency models and may have utility for other types of screens. All of these currently unexplored screening methods hold the possibility of identifying clinically relevant host factors for future validation and potential therapeutic use.

Given the critical importance of cell type in screen design, leveraging CRISPR-Cas9 in primary cell models holds the potential to unlock many novel insights into HIV-1 host factor biology. While advances in CRISPR-Cas9 editing of primary cells have opened the door for CRISPR screens in primary cells models of HIV-1 infection, there have been only limited attempts to leverage these technologies for the purposes of screening. New technologies for pooled CRISPRko, CRISPRi, and CRISPRa screening in primary CD4+ T cells should enable more of these studies in the future. Given that HIV-1 is known to infect myeloid cells and that protocols for editing these cells via CRISPR-Cas9 have now been established [58], conducting screens in these cell types will additionally lead to new understandings surrounding the complex interactions between HIV-1 and the diverse cell types it infects in the body [60].

## 3. Application of CRISPR-Cas9 Gene Editing in HIV-1 Research and Therapy

Beyond its use in genetic screening, CRISPR-Cas9 has also enabled scientists to efficiently validate and mechanistically interrogate complex cellular processes at the host–pathogen interface. In this section, we aim to highlight several studies that have utilized CRISPR-Cas9 systems to better understand the vast network of host–pathogen interactions that drive HIV-1 infection and pathogenesis. Additionally, we discuss how these efforts are being leveraged towards the development of next-generation HIV therapies.

### 3.1. Disruption of HIV-1 Co-Receptors

Many applications of CRISPR-Cas9 host factor editing with the goal of long-term control of HIV-1 infection have focused on targeting the co-receptors used by HIV-1 during viral entry. The HIV-1 replication cycle begins with attachment of the virus to the host cell surface, followed by fusion of the viral and cell membranes. Attachment and fusion are mediated by the viral glycoprotein Envelope (Env) that is embedded in the virion outer membrane. Upon encountering a target cell, Env binds to its primary receptor, CD4, which induces a conformational change in Env that allows for co-receptor binding [61]. Depending on viral tropism, HIV-1 viruses may use one of two co-receptors, CXCR4 or CCR5. Upon co-receptor binding, the gp41 fusion peptide of Env is exposed and inserted into the host cell membrane, ultimately leading to fusion and deposition of the viral core into the host cell cytoplasm [62,63]. Blocking the interaction of Env with these receptors has been heavily explored for the development of novel therapeutic, preventative, and even curative strategies. Indeed, several entry inhibitors have been approved by the FDA for the treatment of HIV-1 infection, and multiple clinical trials are underway on the use of broadly neutralizing antibodies in long-lasting therapeutic or preventative formulations [64]. While pharmacologic or biologic inhibitors are effective, they require continuous treatment in order to maintain the block to entry. As such, genetic disruption of the co-receptors to permanently prevent viral entry has also been explored as an alternative strategy to inhibit HIV-1 infection, especially in the context of a cure [65,66,67].

**Figure 3:**
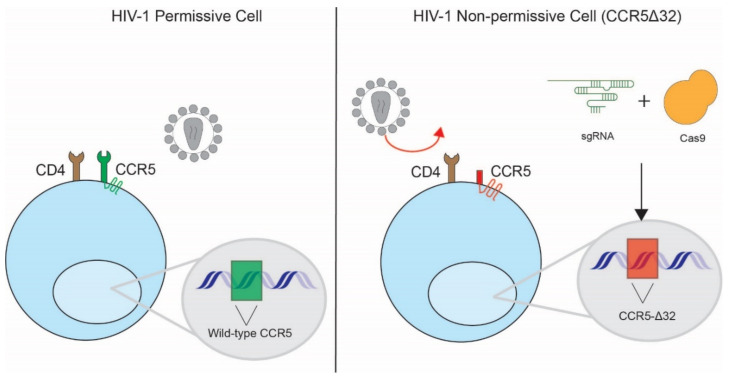
**Induction of CCR5-Δ32 into CD4+ T cells using CRISPR Cas9.** HIV infects cells through engagement of its main receptor, CD4, and a co-receptor, such as CCR5. A minor allele of *CCR5* containing a 32 base pair exonic deletion results in the expression of a truncated protein that is unable to engage HIV Env and mediate viral entry. CRISPR-Cas9 gene editing has enabled the engineering of CD4+ T cells and stem cells that carry this mutation for use in curative cell-based therapies.

In vivo perturbation of the main HIV receptor, CD4, is not feasible due to the biological significance of CD4 in regulating immune function. However, naturally occurring polymorphisms in the co-receptor, *CCR5*, have been instrumental in conceptualizing HIV-1 cure strategies [68,69,70]. A naturally occurring minor allele of *CCR5* harboring a 32 base pair deletion (*CCR5∆32*) that results in a truncated co-receptor was found to be protective against HIV-1 infection in repeatedly exposed, but uninfected, individuals. In the only two reported cases of HIV-1 cure to date, heterologous transplantation of hematopoietic stem cells carrying this *CCR5Δ32* allele into patients resulted in sustained viral suppression after cessation of antiretroviral therapy [71,72]. While this outcome suggests that hematopoietic stem cell transplantation with HIV-1-resistant cells could serve as a route to a sterilizing or functional cure, application of this strategy is highly limited to specialized populations of persons living with HIV (PLWH) in need of transplantation and for whom compatible donors carrying the *CCR5Δ32* mutation may be found [73] (Figure 3).

To circumvent the obstacle of finding compatible donors that carry this rare genetic mutation, CRISPR-Cas9 technology has been used to edit induced pluripotent stem cells (iPSCs) for potential downstream use in autologous stem cell transplantation. In one of the first studies to demonstrate this possibility, Ye et al. utilized a combination of CRISPR-Cas9 and piggyBAC technology to generate iPSCs homozygous for the *CCR5∆32* allele [74]. Under this approach, CRISPR-Cas9 was used to induce a double-stranded DNA break in the *CCR5* locus where the selectable piggyBAC transposon was inserted by homologous recombination [75]. Subsequent excision of the transposon resulted in an exact recreation of the *CCR5∆32* allele. Importantly, monocytes and macrophages derived from these iPSCs were resistant to CCR5-tropic HIV-1. The persistence of this mutation throughout the differentiation process illustrated the therapeutic potential of CRISPR editing through the fixation of heritable changes [76]. Critically, while zinc finger nucleases or TALEN technology were also used for editing of iPSCs, CRISPR-Cas9 achieved higher editing efficiency and caused fewer off-target effects than these other gene editing approaches [77].

Note that while the *CCR5Δ32* allele has since been linked to weakened immunity to other pathogens, alternate protective alleles with fewer detrimental effects may serve as the basis for future strategies [71,78]. Relying on the generation of stochastic insertions and deletions at the CRISPR-Cas9 cut site, Kang et al. reported *CCR5* editing efficiencies of 12.5% with single gRNA and 27% using two gRNA in human iPSCs [66]. After cell sorting to establish clonal iPSC lines, they were able to differentiate these cells successfully into hematopoietic cells carrying diverse *CCR5* mutations. These cells remained susceptible to HIV-1 infection by CXCR4-tropic strains but were resistant to challenge with CCR5-tropic strains, suggesting that their editing was *CCR5*-specific and did not overtly alter cell viability. A number of repair outcomes were sufficient for protection, many of which have not been observed in the human population. Determining the least invasive mutation that provides the strongest and long-lasting protection to infection remains a critical challenge.

Rather than directly targeting the co-receptor itself, host factors that regulate co-receptor expression have been explored as alternate therapeutic targets for preventing HIV-1 infection. For example, Park et al. identified SLC35B2 and TPST2 as HIV host factors in a genome-wide CRISPR screen [22]. Both these proteins are required for sulfonation of host proteins at the cell surface. SLC35B2 transports activated sulfate donors into the Golgi apparatus where TPST2 acts to transfer the sulfate onto protein targets. The N-terminus of CCR5 is sulfonated and this modification is required for its interaction with HIV-1 Env [79]. Indeed, while CRISPR-Cas9-mediated knock-out of TPST2 and SLC35B2 expression in primary CD4+ T cells did not influence the total levels of CCR5 on the cell surface, it dramatically reduced the levels of sulfonated CCR5. This resulted in dramatic decreases in susceptibility to HIV-1 infection, phenocopying *CCR5* knock-out itself. While sulfonation is a widely used post-translational modification for regulating proteins at the cell surface, other regulatory pathways that may be more specific to CCR5 might be more amenable for therapeutic targeting.

In addition to *CCR5*, CRISPR-Cas9 has been used extensively to target *CXCR4* and generate cells that are resistant to CXCR4-tropic HIV-1 infection in vitro. This has included editing in multiple T cell line models as well as in primary CD4+ T cells from both humans and rhesus macaques [80,81]. While a useful control for virological assays in vitro, the potential for *CXCR4* editing as a therapeutic strategy is limited. In the course of natural infection, transmitted founder viruses are exclusively CCR5-tropic, with tropism switching only occurring in some patients after long-term infection [82]. While it is still unclear what role CXCR4-tropic viruses may play in disease pathogenesis, targeted CXCR4 depletion would be insufficient to prevent transmission and is unlikely to succeed as a curative strategy.

### 3.2. Modification of HIV-1 Dependency Factors

**Figure 4:**
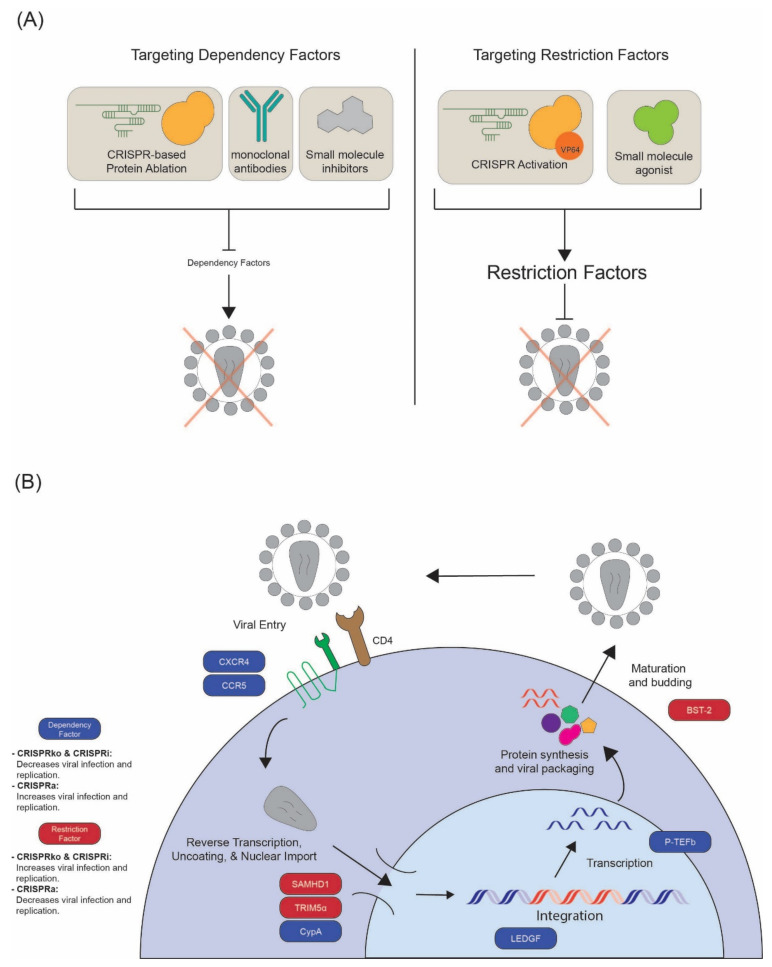
**HIV-1 host factors influence the viral life cycle.** (**A**) Through genetic perturbation or chemical inhibition, researchers can decrease the expression or activity of a given HIV dependency factor to reduce HIV infection and replication. Likewise, through the use of gene activation or small molecule agonists, researchers can increase protein expression or activity of restriction factors to reduce HIV infection. The font size of host “dependency” and “restriction” factors demonstrate how the expression level of these proteins affect viral infection levels. (**B**) Throughout the course of the viral life cycle, HIV encounters several host proteins, some shown here. Certain proteins, which are recruited to assist in the various stages of infection, are known as dependency factors (in blue) while others are known to restrict HIV infection (in red).

Besides the receptor and co-receptors, HIV is dependent on a large number of host proteins at each stage of its lifecycle to achieve successful replication. Modulation of these dependency factors has been shown to hamper HIV-1 infection and replication in vitro, several of which may serve as novel targets for therapeutic intervention. As discussed previously, the advent of CRISPR-Cas9 screening technologies has enabled discovery of several new factors, while targeted application of CRISPR-Cas9 in primary cell types and animal models has improved our understanding of host factor function (Figure 4). This has already informed the mechanism-of-action of new antiretroviral drugs and has suggested new avenues for therapeutic development.

One of the newest drug targets for therapeutic intervention has been the HIV viral core, which is deposited into the host cell cytoplasm following membrane fusion. The viral core is a protein shell made up of thousands of HIV capsid proteins that functions to protect the viral RNA genome during reverse transcription and transport to the nucleus [83]. As such, the capsid protein serves to orchestrate multiple stages of early infection, including uncoating [84], reverse transcription [85], and nuclear import [86], each stage of which is dependent on the recruitment of different host proteins. One of the very first HIV host factors described was cyclophilin A (CypA), which binds to capsid in part to prevent premature uncoating which would restrict infection [84,86]. Using CRISPR-Cas9 gene editing in Jurkat T cells and primary CD4+ T cells, it was shown that CypA is recruited to viral cores to prevent recognition and restriction by the antiviral factor TRIM5⍺. Indeed, knock-out of TRIM5⍺ restores the infectivity of HIV when CypA is knocked out or inhibited. The new FDA-approved antiretroviral drug Lenacapavir likewise acts to alter core stability and inhibit nuclear import of the viral genome. While the full mechanism-of-action is still being explored, CRISPR-Cas9 gene editing of the host factor CPSF6, which binds to the core at the same site as Lenacapavir, has suggested that this host factor is not involved in the drug’s restrictive activity [87]. The use of CRISPR-Cas9 to edit host factors involved in uncoating and nuclear transport towards the identification of therapeutic strategies remains a major goal in HIV molecular virology.

One of the major benefits of the CRISPR-Cas9 editing for gene knock-out is the ability to completely ablate protein expression and assign roles for host factors required at only a small fraction of their steady-state levels. For example, the dependency factor LEDGF (p75) is bound by the HIV-1 pre-integration complex (PIC) to tether it to the chromatin and target integration towards areas of active transcription. LEDGF has a long half-life and only small amounts are sufficient to direct proviral integration, so initial attempts to characterize its functional roles using RNAi required optimization of a stable, highly efficient knock-down strategy. CRISPR-Cas9 knock-out of LEDGF, on the other hand, enabled ready detection of an HIV replication defect in primary CD4+ T cells. Nevertheless, the cellular role of LEDGF in tethering host complexes to chromatin excludes LEDGF knock-out as a viable therapeutic strategy. To overcome this, Lampi et al. optimized a CRISPR-Cas9 strategy to induce a specific site-directed mutation, D366N, into *LEDGF,* which disrupted its interaction with HIV-1 integrase but otherwise allowed for normal interaction with host cellular binding partners [88]. This change was sufficient to inhibit, but not entirely prevent HIV-1 replication in the edited cell population.

As illustrated by the examples above, translating dependency factors into targets for host-directed therapies remains a substantial challenge. First, unlike the receptor/co-receptor interactions, few other dependency factors appear to be absolutely required for viral replication in vitro, but rather promote optimal replication. While the virus may rely on one dominant route to infection, host factor redundancies and flexibility in the route to infection may limit the effectiveness of inhibiting any one given dependency factor. It is unclear how these partial blocks to replication would manifest in vivo. Second, many of the known dependency factors are central players in host cell function and so are unlikely to be viable targets in a therapeutic strategy. For example, transcriptional elongation at proviral integration sites is promoted by recruitment of the positive transcription elongation factor b (P-TEFb) complex to sites of nascent transcription by the viral Tat protein [89]. While P-TEFb recruitment by Tat substantially promotes successful replication, P-TEFb is a master regulator of RNA polymerase II [90] and therapeutic targeting by gene ablation would have broad, non-specific impacts. Small molecules or targeted genetic changes that disrupt host–virus interactions, on the other hand, may be more precise, but likewise are much more challenging.

### 3.3. Modification of Host Restriction Factors

While dependency factors promote viral replication, host restriction factors act to inhibit viral replication through a wide variety of mechanisms (Figure 4). Restriction factors may act specifically to inhibit one type of virus or may act more broadly against multiple virus families. In order to successfully replicate, viruses in turn have developed a number of accessory proteins that counteract restriction factor function. For example, the HIV-1 accessory proteins Vif and Vpu and the HIV-2 accessory protein Vpx act to bind and degrade/inactivate the antiretroviral restriction factors APOBEC3G, BST2/Tetherin, and SAMHD1, respectively. As a consequence of this host–pathogen dynamic, host restriction factors often show positive selection signatures in interaction interfaces over evolutionary time. The inability of a virus to overcome the restriction factors of a different species is often a major barrier to zoonotic infection, and polymorphisms within human restriction factors have even been observed to select for specific viral variants that can escape that restriction. Given the known role of restriction factors in blocking viral transmission, therapeutic avenues to either boost restriction factor expression or prevent viral counteraction have long been discussed.

As mentioned above, one of the first restriction factors encountered by the HIV-1 following cell entry is TRIM5⍺. TRIM5⍺ assembles onto the capsid lattice of incoming cores and induces premature uncoating [91]. This results in inhibition of reverse transcription and nuclear import. To counteract TRIM5⍺, HIV-1 capsid recruits CypA, which masks incoming cores from TRIM5⍺ binding. CRISPR-Cas9-mediated knock-out or chemical inhibition of CypA thus results in strong blocks to HIV-1 infection, which can be rescued by knock-out TRIM5⍺ in primary CD4+ T cells [84]. Rhesus macaque TRIM5⍺ is more efficient at inhibiting HIV-1 despite the presence of CypA, in part due to an R335G mutation that enhances binding to capsid. Desailiniers et al. [92] used CRISPR-Cas9-mediated homology-directed repair to introduce the R335G mutation into the endogenous human *TRIM5*⍺ locus in the Jurkat T cell line. One isolated clone containing the R332G mutation in both alleles significantly enhanced protection against HIV-1 infection and exhibited a ~40-fold increase in antiviral activity.

As opposed to specific mutations that boost restriction factor activity, strategies to boost restriction factor expression have also been explored using CRISPRa. For example, Bogerd et al. [93] used a CRISPRa strategy to induce expression of the restriction factor APOBEC3G (A3G). The APOBEC family of proteins are cellular polynucleotide cytidine deaminases, several members of which potently inhibit HIV-1 replication through direct inhibition of reverse transcription as well as hypermutation of newly synthesized reverse transcripts [94]. To overcome this restriction block, HIV-1 encodes an accessory protein known as Vif, which acts as a substrate adapter between the APOBEC3 proteins and a CUL5-RING E3 ubiquitin ligase that targets the APOBEC3′s for degradation. While Vif is effective at decreasing the steady-state level of the restrictive APOBEC3 family members, including A3G, this can be overcome through overexpression. Increasing expression of A3G using a CRISPRa strategy was sufficient to inhibit both *vif*-deficient as well as wild-type HIV-1 in cell models of infection [93]. Similar results were observed using CRISPRa to increase expression of the restriction factor BST-2/Tetherin [95]. BST-2/Tetherin prevents the budding of mature HIV-1 viral particles by physically tethering them to the producer cell. To overcome this block, HIV-1 encodes Vpu, which removes BST-2/Tetherin from the cell surface and induces its degradation. Zhang et al. demonstrated that CRISPRa to increase BST-2/Tetherin expression was sufficient to inhibit HIV-1 replication even in the presence of Vpu [96].

Several host restriction factors have been interrogated in T cells, but there are a number of restriction factors that play unique roles in macrophages and other infected myeloid cells. For example, the restriction factor SAMHD1 acts to inhibit HIV infection of resting T cells and macrophages by the depletion of dNTPs and the subsequent inhibition of reverse transcription [97,98]. To overcome this block, HIV-2 and numerous SIVs encode the accessory protein Vpx, which binds to and induces the degradation of SAMHD1 [99,100]. Indeed, to achieve infection of monocyte-derived macrophages in vitro with HIV-1, which does not encode Vpx, research teams will often deliver SIV Vpx exogenously to degrade SAMHD1 before challenge. Using a new approach to generate polyclonal knock-out pools of primary monocytes, Hiatt et al. recently demonstrated efficient knock-out of SAMHD1 [58]. After differentiation into macrophages, the SAMHD1 knock-out cells exhibited 50-fold increased infection with HIV-1. Taken together, strategies to boost restriction factor activity show promise given their known roles in restricting viral transmission, but the many ways in which the virus has overcome these blocks in the past suggests a continued ability to evolve resistance in a therapeutic setting.

### 3.4. Modification of Host Immune Factors

Most described restriction factors are interferon-stimulated genes (ISGs) that act as part of the innate immune system to inhibit viral infection. Upon infection, host proteins known as pattern recognition receptors (PRRs) act to specifically recognize conserved pathogen-associated molecular patterns (PAMPs), and subsequently initiate a signaling cascade that triggers the interferon response and ISG expression. Depending on the cell type and the PAMP that is sensed, different ISGs are expressed under different circumstances. The collective activity of the expressed ISGs creates a less permissive environment for a broad array of pathogens. Indeed, recombinant interferon has sometimes been administered as a therapeutic agent in circumstances where specific antivirals may not be available, i.e., in the treatment of hepatitis B virus [101] before the advent of effective nucleoside analogs or in the treatment of AIDS-associated Kaposi’s sarcoma herpesvirus [102].

While the innate immune response to HIV-1 is still being characterized, several groups have explored CRISPR-Cas9 as a tool to map the PRRs that sense infection and to identify the ISGs that directly impact HIV-1 replication. While RIG-I/MDA5 [103,104] and cGAS/IFI16 [105,106,107] are thought to be the primary innate immune sensors of HIV-1 RNA and DNA, respectively, a number of new factors that modulate this response have since been described. For example, Lahaye et al. [108] found that NONO, a direct interactor of HIV-1 capsid, increases cGAS-mediated immune activation during productive HIV-1 infection. Knock-out of NONO using CRISPR-Cas9 reduced the innate immune response to infection through the cGAS signaling axis. On the other hand, other proteins have been discovered that act to suppress the host immune response during HIV-1 infection, including PLK1, which dampens the type I interferon response generated downstream of MAVS signaling [109]. A number of other studies using CRISPR-Cas9 to characterize the role of other ISGs in the antiviral response are ongoing. For example, as detailed earlier, OhAinle et al. leveraged an ISG CRISPR library termed PIKA-HIV to identify TRIM34 as a novel restriction factor acting alongside TRIM5⍺ to induce premature uncoating [29]. Overall, while modulation of the innate immune response to temper acute infection and/or regulate inflammation has proven therapeutically tenable, these efforts are likely to be limited to the provision of immune modulatory agents as opposed to gene-based therapies. That being said, as cell-based therapies are advanced for the treatment of diverse human diseases, genetic manipulation of these cells’ innate immune pathways to either prevent infection or trigger a specific immune state may be explored for added benefit.

### 3.5. HIV-1 Latency

One of the largest obstacles to an HIV cure is the persistence of the virus in long-lived cellular reservoirs. These reservoirs of latently infected cells evade immune clearance and can reseed infection upon ART cessation. While a large number of groups have investigated the use of CRISPR-Cas9 systems to directly target and excise integrated HIV-1 proviruses in latently infected cells [110,111,112], here we will focus on the use of CRISPR-Cas9 to modulate host factors that influence HIV latency establishment and maintenance.

HIV latency is mediated by several mechanisms that inhibit successful viral transcription, including epigenetic blocks, blocks to transcriptional initiation, blocks to transcriptional elongation, and blocks to transcriptional termination and RNA processing. These overlapping mechanisms can vary by cell type, cell state, as well as by proviral integration site, complicating their study. As highlighted above, several genome-wide knock-out studies have been performed in cell line models of HIV-1 latency to discover host factors that may contribute to latency reversal and maintenance. These have identified a number of host factors involved in the regulation of viral transcription. For example, P-TEFb is recruited to RNA Pol II at sites of nascent proviral transcription by the HIV-1 Tat protein to license transcriptional elongation. Ma et al. showed that P-TEFb’s catalytic subunit, CDK9, is SUMOylated by TRIM28 [113], inhibiting P-TEFb assembly and viral gene transcription. Using CRISPR-Cas9, they determined that knockout of TRIM28 resulted in increased transcriptional activity at HIV-1 promoter regions, suggesting that P-TEFb regulators may be candidate therapeutic targets for modulating latency. Knock-out of P-TEFb itself heavily restricts viral replication and has been explored as a therapeutic target, though this is not likely tenable given the central role of P-TEFb in cellular transcriptional regulation [31].

While these cell line-based models of latency have been effective tools for understanding proviral transcription, it is unclear how translatable these findings are to latent reservoirs in PLWH. A number of primary cell models have been developed to reflect the latent state, but even these yield highly discordant results [114] and often do not recapitulate the full physiological picture of HIV latency [115]. Cells from PLWH who have achieved viral suppression on ART are considered the gold standard for studying the latent state, but even these cells may not faithfully represent the latent reservoir given they are nearly always derived from the blood (not the tissue), often require ex vivo culturing before analysis, and usually harbor very few latently infected cells. What is more, the use of CRISPR-Cas9 in most primary cell latency models and in latently infected cells from PLWH has been exceedingly difficult. For example, resting memory CD4+ T cells, one of the major components of the latent reservoir [116,117,118,119,120], have proven difficult to edit for a number of reasons, including their closed chromatin structure and slow rate of protein turnover [121]. For this reason, researchers have relied on the use of small molecule inhibitors of target proteins to understand their function in HIV latency, though these are prone to off-target effects. Significant strides have been made recently to develop more robust protocols for CRISPR-Cas9 gene editing in resting CD4+ T cells [122], but the development of compatible latency assays are still required to significantly advance the field.

### 3.6. Considerations and Future Directions of CRISPR Therapies

CRISPR-Cas9 gene editing has proven to be a powerful and versatile tool with applications throughout the therapeutic pipeline from target discovery to validation and application. The ease and accessibility of whole genome screening across a variety of model systems has revolutionized the small molecule drug discovery pipeline and has uncovered a variety of novel drug targets for a myriad of diseases [123]. It has likewise greatly facilitated a number of cell-based therapeutic strategies, from the creation of de novo chimeric antigen receptor T cells for the treatment of cancer to the manipulation of hematopoietic stem cells for immunotherapy. Multiple studies are underway to examine the potential for CRISPR-Cas9 technology to generate the *CCR5Δ32* mutation ex vivo to enable autologous transplant of HIV-1-resistant cells.

The use of CRISPR-Cas9 for the in vivo correction of in-born genetic defects or to attack drug-resistant pathogens or cancers has likewise been widely touted, though there are still a wide array of limitations and ethical considerations that must still be addressed as this technology advances. One of the overarching limitations to conceptualizing the use of CRISPR-Cas9 as a therapeutic in vivo is the difficulty of delivering the complexes to the right place in the body at the right time. Viral-based vectors such as adenoviral, adenoviral-associated, and lentiviral vectors can be used to deliver gRNA to target cells, but their application has been limited, and there has been mixed clinical success [124]. Furthermore, the safety of viral-based vectors in specialized patient subsets with dysfunctional immune responses, such as PLWH, is highly debatable. Even if it could be delivered efficiently and safely, one of the persistent issues with CRISPR-Cas9 gene editing as a therapeutic has been the potential for off-target effects. Several of the studies referenced in this review showed that limited off-target cleavage occurs in their models, but it is still unknown what level of off-target risk might be ‘acceptable’ in a therapeutic context. Proposed engineering strategies to overcome this risk include the delivery of a one-time payload of Cas9 RNPs to limit the opportunity for off-target editing. In the context of HIV, groups have developed alternate strategies, such as cloning the Cas9 gene under the HIV-1 promoter to limit Cas9 expression to Tat-expressing cells, but this could be difficult to scale up to a clinically relevant therapeutic strategy [125].

While there are still a large number of limitations, it has only been a decade since it was first demonstrated that CRISPR-Cas9 could be used for precision editing of the human genome. Since then, this technology has revolutionized biomedical research, uncovering biological pathways that underlie diverse human diseases and unveiling new high-priority drug targets. With continued advances in the technology, we now stand at the cusp of a new era of genetic medicine with broad applications for fighting a diverse array of human disease. The use of CRISPR-Cas9 gene editing in HIV host factor discovery and validation has advanced the field and will undoubtedly inform the development of next-generation, host-directed therapeutic strategies in the decades to come.

## Figures and Tables

**Table 1:** **HIV-1 Host Factor Screens.** Previously conducted host factor screens using both RNAi and CRISPR-Cas9 technologies. Hits: Number of genes determined to be significant in process of interest in screen. Genes Targeted: Number of total genes targeted for genetic perturbation in screen. Hit Rate: Percent of targeted genes identified as screen hits. Gene List Description: Description of composition of library used in screening. Technology: Gene editing technology used for genetic perturbation. Pooled or Arrayed: Designates whether screen was conducted in arrayed or pooled format. Cell Type: Cell type into which screening library was delivered. Readout: Reporter or phenotype assessed to determine screen hits. Virus Used: Virus used for infection of cells used in screen. For screens involving the use of latently infected cells, the sequence of the harbored provirus within the cell is listed. Genes of Interest: Categories of host factors of interest for discovery in screen. Screen hits: Selected host factors identified in the screen and validated/highlighted by the authors.

Study	Hits	Genes Targeted	Hit Rate	Gene List Description	Technology	Pooled or Arrayed	Cell Type	Readout	Virus Used	Genes of Interest	Screen Hits
Yeung [18]	252	54,509	0.46%	54,509 transcripts, 3–5 shRNA per gene	shRNA	Pooled	Jurkat	Cell survival	HIV (NL4-3)	HIV replication	NRF1, STXBP2, NCOA3, PRDM2, EXOSC5
Brass [19]	386	21,121	1.83%	Genome wide, 21,121 pools of 4 siRNAs per gene	siRNA	Arrayed	TZM-bl	p24 Immunofluorescence, β-galactosidase	HIV (IIIB)	HIV replication	Med28, TNPO3, Rab6, VPS53
König [20]	295	20,000	1.48%	Genome wide, targeting 20,000 genes	siRNA	Arrayed	HEK293T	Luciferase	HIV (VSV-pseudotyped HIV-1-luciferase vector)	HIV replication	TNPO3, NUP153, NUP358
Zhou [21]	311	19,709	1.58%	Genome wide, targeting 19,709 genes, pools of 3 siRNAs per gene	siRNA	Arrayed	HeLa P4/R5	β-galactosidase	HIV (HXB2)	HIV replication	AKT1, PRKAA1, CD97, NEIL3, BMP2K, SERPINB6
Park [22]	5	18,543	0.03%	Genome wide, 187,536 sgRNAs targeting 18,543 protein-coding human genes	CRISPR	Pooled	GXCRCas9 T cell line	Cell survival	CCR5-tropic HIV-1 strain JR-CSF	Host dependency factors	TPST2, SLC35B2, ALCAM
Huang [23]	446	3733	11.9%	sgRNA libraries enriched with human nuclear proteins	CRISPR	Pooled	J–LAT A2	GFP expression	Latently infected ‘LTR-Tat-IRES-GFP’ HIV-1 minigenome’ proviral sequence	Latency	MINA53
Krasnopolsky [24]	5	19,052	0.03%	Genome-scale CRISPR Knock-Out (GeCKO) whole genome library, 19,052 genes, 6 sgRNA constructs per gene	CRISPR	Pooled	Jurkat (2D10)	BFP expression	HIV-LTR-2dGFP proviral sequence	Latency	ZNF304
Li, 2019 [25]	6	20,000	0.03%	Whole genome sgRNA library containing a total of ~200,000 sgRNAs at an average of 10 per gene	CRISPRi	Pooled	Jurkat (2D10-CRISPRi)	GFP expression	HIV-LTR-2dGFP proviral sequence	Latency	PSMD1, NFKBIA, CYLD, GON4L, PSMD3, PSMD8
Li, 2020 [26]	4	20,000	0.02%	~100,000 sgRNA sequences, 5 sgRNAs/gene	CRISPRi	Pooled	JiL cell lines	GFP expression	HIV-LTR-2dGFP proviral sequence	Latency	FTSJ3, TMEM178A, NICN1
Pedersen [27]	18	18,905	0.095%	Genome-wide CRISPRi library, 5 sgRNA per gene	CRISPRi	Pooled	HIV-1-d6-GFP-Jurkat	GFP expression	HIV-1-d6-GFP proviral sequence	Latency	SLTM, SRRM2, NCBP2
OhAinle, 2018 [28]	15	1905	0.79%	1905 human ISGs, 4 sgRNAs per gene	CRISPR (PIKA-HIV)	Pooled	THP-1	Newly budded viruses	PIKA-HIV	ISGs, dependency factors	MxB, TRIM5α, IFITM1, Tetherin
OhAinle, 2020 [29]	9	1905	0.47%	1905 human ISGs, 4 sgRNAs per gene	CRISPR (PIKA-HIV)	Pooled	THP-1	Newly budded viruses	HIV capsid mutants P90A, N74D	Capsid targeting restriction factors	TRIM34
Hultquist [30]	8	45	17.78%	146 crRNAs	CRISPR RNPs	Arrayed	Primary human CD4+ T cells	GFP expression	HIV (NL4-3)	Genes associated with HIV integrase	GEMIN2, KPNA1, KPNA5, XRCC6
Hiatt [31]	47	426	11.03%	HIV-1 Interactome	CRISPR RNPs	Arrayed	Primary human CD4+ T cells	GFP expression	HIV (NL4-3)	Viral restriction and dependency factors	HUWE1, ELOC, AFF1

## Data Availability

Not applicable.
